# AI-Guided Cognitive Behavioral Therapy for Depression and Anxiety: Bridging the Mental Health Treatment Gap Through Digital Psychiatry

**DOI:** 10.3390/healthcare14152334

**Published:** 2026-08-01

**Authors:** Aleksandra Stojanovic, Miodrag Stankovic, Aleksandra Ristic

**Affiliations:** 1 Center for Mental Health Protection, University Clinical Center Nis, 18000 Nis, Serbia; 2 Faculty of Medicine, University of Nis, 18000 Nis, Serbia

**Keywords:** digital mental health, artificial intelligence, cognitive behavioral therapy, depression, anxiety, digital psychiatry, conversational agents, therapeutic alliance, stepped care, treatment gap

## Abstract

**Background:** Depression and anxiety disorders remain among the leading contributors to global disability and represent a major public health challenge. Although evidence-based psychotherapies are available, access to treatment remains limited due to structural, economic, geographical, and workforce-related barriers. Digital mental health interventions have emerged as scalable approaches to reducing this treatment gap, with artificial intelligence (AI)-guided cognitive behavioral therapy (CBT) representing a rapidly developing and clinically relevant extension of digital psychotherapy. **Objective:** This review aims to synthesize current evidence on digital and AI-guided CBT interventions for depression and anxiety, with a focus on clinical utility, scalability, mechanisms of change, safety considerations, and public health relevance. In addition, the review proposes a clinically oriented conceptual framework for understanding the role of AI-guided CBT within contemporary digital psychiatry. **Methods:** A focused narrative review was conducted using PubMed, Scopus, and Google Scholar databases, covering publications from 2010 to 2025. Relevant peer-reviewed studies, systematic reviews, meta-analyses, and conceptual papers addressing digital CBT, AI-assisted CBT, conversational agents, symptom monitoring, and digital mental health implementation were identified and analyzed qualitatively. **Results:** Existing evidence suggests that internet-delivered CBT, mobile applications, and AI-based conversational agents may reduce depressive and anxiety symptoms, particularly in individuals with mild to moderate conditions. However, the evidence base remains heterogeneous, with limitations including short follow-up periods, variability in intervention quality, reliance on self-reported outcomes, and insufficient data on long-term effectiveness, safety, and real-world implementation. Emerging concepts such as digital therapeutic alliance, continuous symptom monitoring, adaptive intervention delivery, and AI-driven personalization may represent key factors influencing engagement and clinical outcomes. **Conclusions:** AI-guided CBT represents a promising but still evolving component of modern mental health care. These technologies have the potential to improve accessibility, optimize resource allocation, and support stepped-care and hybrid models of treatment. Future research should prioritize rigorous clinical validation, long-term outcome evaluation, transparent safety protocols, ethical governance, and integration into real-world health systems. AI-guided CBT should not be understood as a replacement for clinicians, but as a complementary and scalable extension of evidence-based psychotherapy.

## 1. Introduction

### 1.1. Global Mental Health Context

Depression and anxiety disorders represent a major challenge for contemporary mental health care systems. They are among the most prevalent mental disorders worldwide and contribute substantially to the global burden of disease. Beyond their clinical impact, these conditions are associated with reduced quality of life, impairments in social and occupational functioning, and increased health care utilization, resulting in significant societal and economic costs. According to the World Health Organization, depression and anxiety affect hundreds of millions of people globally, underscoring their importance as leading public mental health concerns [1,2].

Although evidence-based psychotherapies are available, access to treatment remains limited across many settings. Cognitive behavioral therapy (CBT) is one of the most empirically supported first-line psychotherapeutic approaches for depression and anxiety [3,4,5]. However, the availability of effective treatments does not guarantee their accessibility. Persistent barriers include shortages of trained mental health professionals, long waiting lists, financial constraints, geographical limitations, and stigma associated with seeking help [6,7].

These factors contribute to the so-called “mental health treatment gap,” which has become a central concept in global mental health. Addressing this gap has emerged as a key priority, prompting increasing interest in scalable, cost-effective, and accessible models of care. In this context, digital mental health interventions have gained attention as promising tools for expanding access to psychological support while maintaining alignment with evidence-based therapeutic principles.

### 1.2. Digital Mental Health Interventions and Artificial Intelligence in Psychotherapy

Digital mental health interventions encompass a wide range of approaches, including teletherapy, guided self-help programs, internet-delivered CBT, mobile applications, and more recently, AI-enabled systems. Their growing appeal lies in their ability to deliver psychological support in flexible and accessible formats, often outside traditional clinical settings, thereby reducing practical barriers to help-seeking and treatment adherence.

Over the past decade, the evidence base for digital interventions has expanded substantially, particularly in the treatment of depression and anxiety. Internet-delivered CBT, in particular, has demonstrated outcomes comparable to face-to-face therapy in certain patient groups and under specific conditions, especially when guided and structured [8,9].

The integration of artificial intelligence has further extended the potential of these interventions. AI-guided psychotherapy systems are designed to provide structured support, respond interactively to user input, monitor symptom changes, and adapt content based on user behavior and needs. These systems include text-based chatbots, voice-based conversational agents, passive monitoring tools, generative AI interfaces, and adaptive digital therapy platforms. Their potential lies in offering personalized, continuously accessible support at scale, with reduced reliance on therapist time [10,11,12,13,14]. However, AI-guided psychotherapy does not possess the clinical judgment, emotional understanding, and contextual sensitivity of a trained mental health professional. For this reason, its role is best understood as complementary rather than substitutive. At the same time, rapid advances in AI technology have raised important questions about how these systems may extend the reach of psychological treatment, particularly in the context of highly prevalent conditions such as depression and anxiety.

### 1.3. Rationale, Novelty, and Aim of the Review

The focused evaluation of AI-guided psychotherapy is supported by several converging developments. Digital mental health interventions have already established a foundation for structured, remote, and self-directed therapeutic care. Building on this foundation, AI systems introduce additional capabilities, including interactive communication, personalization, continuous support, and real-time adaptation based on user behavior.

These features may enhance engagement, adherence, and responsiveness of interventions, particularly through mechanisms such as adaptive feedback, symptom monitoring, and data-driven tailoring of therapeutic content. At the same time, the scalability of such systems creates the potential to reach large populations who might otherwise remain untreated.

However, the current body of evidence remains limited and methodologically heterogeneous. Existing studies often rely on small sample sizes, short intervention durations, self-reported outcomes, and diverse technological platforms with varying levels of therapeutic structure. In addition, many available tools have not been adequately evaluated in real-world clinical settings. These limitations make it difficult to draw firm conclusions regarding efficacy, safety, and long-term effectiveness.

Importantly, there is also a lack of conceptual clarity regarding the mechanisms through which AI-guided interventions exert their effects. In particular, the roles of user engagement, digital therapeutic alliance, and structured therapeutic exposure remain insufficiently understood.

This issue has become particularly relevant with the emergence of large language model-based systems, where conversational fluency may create a strong impression of responsiveness, while clinical effectiveness, safety, and mechanisms of change remain insufficiently established [13,14,15].

The novelty of this review lies in its integrative conceptual approach. Rather than presenting AI-guided CBT only as a technological extension of conventional digital interventions, this paper brings together clinical mechanisms of CBT, AI-driven personalization, digital therapeutic alliance, symptom monitoring, safety considerations, and public health implementation. By integrating these domains, the review proposes a clinically oriented framework for understanding how AI-guided CBT may function as a complementary and scalable component of care for depression and anxiety disorders.

The aim of this paper is to provide a focused synthesis of current evidence on AI-guided cognitive behavioral therapy for depression and anxiety, integrating clinical and public health perspectives. In addition to summarizing existing findings, this review seeks to identify key challenges, explore emerging mechanisms of action, and propose a conceptual framework to guide future research and implementation of AI-supported psychotherapy.

## 2. Methods: Literature Review Approach

This study was conducted as a focused narrative review of the literature on digital and AI-guided cognitive behavioral therapy (CBT) for depression and anxiety disorders. Although it was not designed as a full systematic review or meta-analysis, elements of a structured search strategy were incorporated to enhance transparency, methodological clarity, and reproducibility of the literature selection process.

A literature search was performed using electronic databases, including PubMed, Scopus, and Google Scholar, covering publications from 2010 to 2025, in order to capture the rapid development of digital mental health interventions, internet-delivered CBT, conversational agents, and AI-supported psychotherapy systems.

The following keywords and their combinations were used: “cognitive behavioral therapy”, “CBT”, “internet-delivered CBT”, “digital mental health”, “digital psychiatry”, “AI-guided therapy”, “AI-guided CBT”, “artificial intelligence”, “chatbots”, “conversational agents”, “machine learning in psychiatry”, “depression”, and “anxiety disorders”.

The search strategy combined CBT-related terms with digital and AI-related terms and diagnostic terms. Examples of search combinations included: “AI-guided CBT” AND depression, “AI-guided CBT” AND anxiety disorders, “digital mental health” AND cognitive behavioral therapy, “chatbots” AND depression, “conversational agents” AND anxiety, “internet-delivered CBT” AND depression, “internet-delivered CBT” AND anxiety, and “machine learning” AND psychiatry AND mental health. A total of 35 sources were included in the final reference list, including empirical studies, systematic reviews, meta-analyses, narrative and scoping reviews, conceptual papers, reports, and methodological sources.

Studies were included if they:

(1) examined digital, internet-delivered, or AI-assisted CBT interventions;
(2) focused on depression and/or anxiety disorders;
(3) reported clinical, implementation, engagement, safety, or conceptual outcomes relevant to digital mental health;
(4) were published in peer-reviewed journals; and
(5) were available in English.

Randomized controlled trials, systematic reviews, meta-analyses, observational studies, real-world evaluations, and relevant conceptual papers were considered in order to provide a comprehensive and clinically relevant overview of the field.

Studies were excluded if they:

(1) focused primarily on non-CBT interventions;
(2) addressed psychiatric conditions outside the scope of depression and anxiety disorders without separate consideration of these conditions;
(3) described purely technical AI models without clinical or psychotherapeutic relevance; or
(4) lacked sufficient methodological clarity.

The selected literature was analyzed qualitatively, with particular attention to intervention characteristics, target populations, clinical outcomes, user engagement, proposed mechanisms of action, ethical and safety considerations, and implications for stepped-care and hybrid models of mental health care.

## 3. Digital and AI-Guided CBT for Depression and Anxiety

### 3.1. Digital Interventions for Depression and Anxiety

Digital interventions for depression and anxiety are increasingly recognized as promising approaches for expanding access to evidence-based care. Among these, internet-delivered CBT (iCBT) represents the most extensively studied modality, with numerous randomized controlled trials demonstrating its effectiveness, particularly for individuals with mild to moderate symptoms. In certain contexts—especially when guided and structured—outcomes may be comparable to those of face-to-face therapy [8,9]. Mobile applications have further extended this model by providing continuous access to psychoeducation, therapeutic exercises, mood monitoring, and behavioral activation techniques. These tools may support ongoing therapeutic engagement and enhance self-management, both between sessions and in fully self-directed formats. In parallel, teletherapy has emerged as an effective method for delivering psychotherapy remotely, with positive outcomes reported across diverse clinical settings [16].

Despite these advantages, digital interventions also face persistent challenges, including variable user engagement, high dropout rates, and heterogeneity in application quality. Recent reviews highlight that effectiveness depends not only on the availability of interventions, but also on usability, user experience, and sustained engagement [17,18].

Taken together, digital mental health interventions provide an important foundation for understanding the emergence of AI-guided psychotherapy. They demonstrate that technology can deliver structured psychological treatment, while also emphasizing that engagement, usability, and sustained interaction are critical determinants of clinical outcomes.

### 3.2. Artificial Intelligence-Guided Conversational Agents in Psychotherapy

One of the most prominent developments within AI-guided psychotherapy is the use of chatbots, or AI-based conversational agents. These systems typically operate through text- or voice-based interfaces and provide psychoeducation, cognitive restructuring prompts, behavioral exercises, emotional support, and symptom monitoring. Many of these interventions are conceptually grounded in cognitive behavioral therapy principles and are designed to simulate structured elements of therapeutic dialogue [10,11,19,20].

Conversational agents offer several practical advantages. Their continuous availability and immediate responsiveness may increase accessibility, particularly for individuals who are reluctant to seek traditional mental health services. In addition, their scalability allows a single system to support a large number of users simultaneously, making them potentially valuable from a public health perspective.

However, the current literature also highlights important limitations. Existing studies are often short-term, rely heavily on self-reported outcomes, and examine systems that vary substantially in design, functionality, and therapeutic structure. Moreover, key questions remain regarding the depth and durability of therapeutic effects, the nature of user engagement, and the extent to which interactions with these systems can approximate meaningful therapeutic processes.

Recent reviews and meta-analyses support this cautious interpretation. Although AI-based and CBT-based chatbots have shown promising short-term effects on depressive symptoms and, to a lesser extent, anxiety symptoms, the evidence remains limited by heterogeneity of interventions, short follow-up periods, and inconsistent reporting of mechanisms and implementation features [19,20].

In particular, the question of whether users develop a form of therapeutic alliance with conversational agents remains unresolved, representing an important area for further investigation.

### 3.3. Monitoring Depressive and Anxiety Symptoms Using Artificial Intelligence

Symptom monitoring represents an important area of AI application in mental health. In traditional psychotherapy, assessment typically relies on periodic questionnaires and clinician judgment based on session-based observations. AI-enabled systems have the potential to extend this process by enabling continuous or repeated symptom tracking through self-report tools, interaction data, and passively collected behavioral indicators.

Standardized instruments such as the Patient Health Questionnaire-9 (PHQ-9) [21] and the Generalized Anxiety Disorder-7 (GAD-7) [22] are frequently used in digital settings. These tools allow repeated assessment of symptom severity over time and may be particularly useful for identifying patterns and trajectories of change, rather than relying solely on intermittent evaluations.

In addition to structured scales, AI systems increasingly incorporate digital phenotyping, which refers to the use of data generated through smartphone use, device interactions, movement patterns, communication behavior, sleep-related signals, or linguistic features to infer changes in mental state [23]. These approaches may enhance clinical insight and support early detection of symptom changes. In the context of newer LLM-based systems, such monitoring may be complemented by analysis of natural language interactions, although the clinical validity and reliability of these approaches require further evaluation [14,15]. They may also contribute to increased self-awareness by helping users recognize patterns in their emotions, thoughts, and behavior.

However, digital phenotyping remains an emerging field. Concerns related to validity, reliability, generalizability across populations, and the ethical use of sensitive data remain significant. Therefore, while AI-assisted symptom monitoring shows considerable promise, it requires further rigorous clinical validation.

Key findings from representative studies on digital and AI-guided CBT are summarized in Table 1.

Table 1 was designed as a representative summary rather than an exhaustive presentation of all included studies. The selected studies were chosen to illustrate major evidence categories relevant to this review, including internet-delivered CBT, AI-based chatbots, broader digital interventions, and real-world conversational agent data.

## 4. Conceptual Framework of AI-Guided CBT

AI-guided cognitive behavioral therapy can be conceptualized as a multi-layered intervention in which technological features, user engagement, and therapeutic processes interact dynamically.

At the core of this model is the interaction between AI system functionality and user experience. Features such as personalization, responsiveness, and continuous availability may enhance user engagement, which in turn influences adherence and therapeutic exposure [8,25].

A central, yet still insufficiently understood component of this process is the concept of digital therapeutic alliance. While distinct from traditional face-to-face therapeutic alliance, it may function as a mediating factor that supports sustained interaction with the system and promotes treatment adherence [26,27].

However, it is important to distinguish between perceived alliance (the subjective experience of being understood or supported) and functional therapeutic mechanisms. It is possible that repeated exposure to CBT techniques—such as behavioral activation and cognitive restructuring—rather than relational depth, represents the primary driver of clinical improvement in AI-guided interventions [5].

These processes are further moderated by individual and contextual factors, including symptom severity, digital literacy, motivation, and the presence or absence of human clinical support.

Ultimately, symptom improvement may result from the interaction between structured CBT content delivery and sustained user engagement, facilitated by scalable digital systems.

A comparative overview of traditional CBT, digital CBT, and AI-guided CBT is presented in Table 2, highlighting key differences in delivery format, personalization, monitoring, therapeutic relationship, and implementation challenges.

This framework highlights the need to move beyond purely technological or outcome-based perspectives toward integrative models that link mechanisms of action, user engagement, and clinical outcomes.

**Figure 1:**
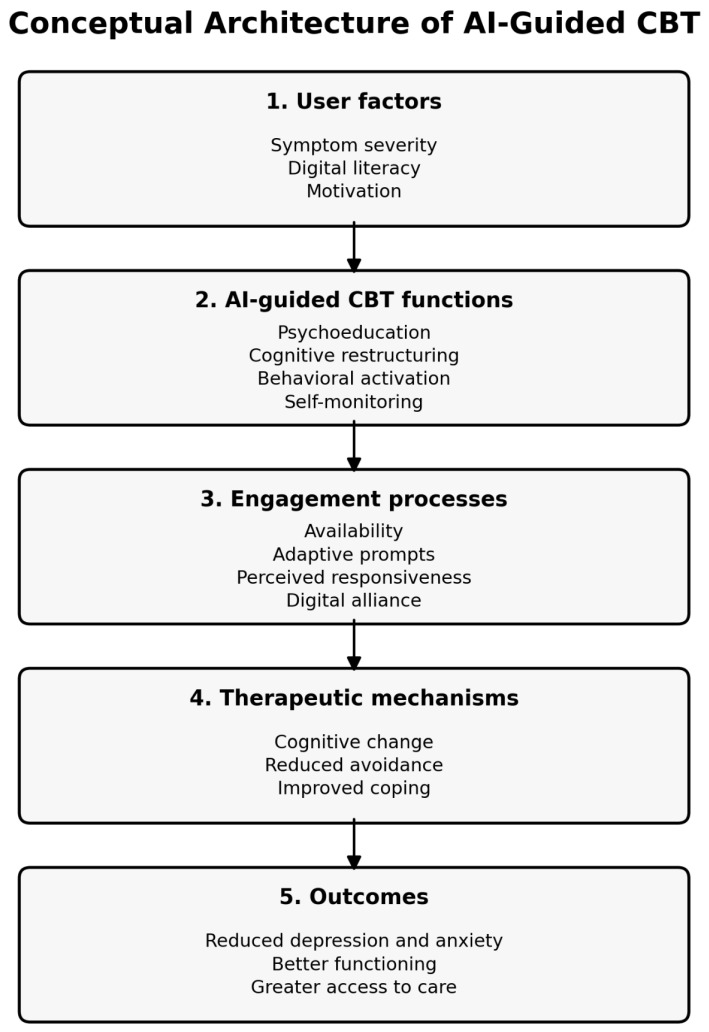
Conceptual architecture of AI-guided CBT for depression and anxiety.

The proposed conceptual architecture of AI-guided CBT is presented in Figure 1. This model illustrates how user characteristics, AI-supported therapeutic functions, engagement mechanisms, and core CBT processes may interact to influence clinical and public health outcomes in depression and anxiety care.

## 5. Public Health, Ethical, and Clinical Implications

### 5.1. Public Health Implications of AI-Guided Psychotherapy

Digital technologies integrated into mental health care are increasingly recognized as potential tools for addressing the mental health treatment gap [23]. AI-guided psychotherapy, in particular, may contribute to reducing pressure on specialized mental health services by supporting psychoeducation, self-help guidance, symptom monitoring, and low-intensity interventions for individuals with mild to moderate symptoms.

This approach aligns with contemporary public health strategies that emphasize prevention, early intervention, and more efficient allocation of resources [28]. By enabling scalable delivery of evidence-based psychological interventions, AI-guided systems may improve access to care, particularly in settings where mental health services are limited.

In addition to expanding access, these technologies may support early detection and intervention, thereby contributing to more effective population-level mental health strategies and potentially reducing the overall burden of depression and anxiety disorders [25,28].

### 5.2. Ethical and Clinical Challenges

Despite its potential, AI-guided psychotherapy raises important ethical and clinical concerns. One of the most prominent issues relates to data security and privacy, as digital platforms often process highly sensitive information, including symptom reports, emotional disclosures, and behavioral patterns. Ensuring robust data protection, meaningful informed consent, and compliance with legal frameworks such as the General Data Protection Regulation (GDPR) is therefore essential for responsible implementation [18,29].

Clinical safety represents another critical consideration. While AI-guided systems may be appropriate for individuals with mild to moderate symptoms, they are not designed to manage complex psychiatric conditions, high-risk presentations, or crisis situations. As a result, human oversight, clear referral pathways, and crisis escalation mechanisms are necessary components of safe and effective use [30].

These concerns are especially relevant for generative AI and large language model-based systems, where fluent and empathic-sounding responses may exceed the level of clinical validation, safety testing, or crisis-management capacity currently available in many commercial tools [13,14].

Algorithmic bias is an additional concern. Systems trained on limited or non-representative datasets may perform less accurately for certain populations, potentially leading to unequal or suboptimal care [31,32]. Addressing these issues requires careful attention to data diversity, transparency in model development, and ongoing evaluation.

Regulatory challenges also remain unresolved, particularly in the context of large-scale implementation. Questions regarding the classification of AI-guided systems, appropriate validation standards, and accountability for adverse outcomes are still under development. These challenges do not negate the potential of AI-guided psychotherapy but underscore the need for cautious governance, transparent system design, and continuous clinical evaluation.

### 5.3. Key Challenges and Future Research Priorities

Despite promising findings, several critical challenges remain. First, the heterogeneity of AI-guided systems complicates comparison across studies, as interventions vary substantially in structure, therapeutic depth, and degree of human involvement. Second, the majority of existing studies focus on short-term outcomes, limiting understanding of long-term effectiveness, relapse prevention, and sustained engagement.

Third, clinical validation in real-world settings remains limited, with many interventions tested in controlled environments or self-selected populations, raising concerns about generalizability. Fourth, ethical and regulatory issues—including data privacy, algorithmic bias, and responsibility for adverse outcomes—require further clarification.

Finally, the role of AI-guided psychotherapy within stepped-care and hybrid treatment models remains insufficiently defined. Addressing these challenges will be essential for advancing the field and ensuring the safe, effective, and equitable integration of AI-guided interventions into mental health care systems.

In light of recent developments in generative AI, future studies should also distinguish between rule-based chatbots, CBT-specific digital therapeutics, and general-purpose large language model systems, as these technologies differ substantially in design, validation requirements, risk profile, and potential clinical use [13,15].

## 6. Discussion

AI-guided psychotherapy appears to occupy a promising but still transitional position between digital self-help tools and therapist-delivered mental health care [12,17]. Its potential advantages include accessibility, scalability, immediacy, and the ability to provide continuous or repeated support [11,18]. These characteristics may be particularly relevant for depression and anxiety, as many individuals face barriers to accessing traditional care yet could benefit from structured, lower-intensity interventions [6,33]. From a public health perspective, AI-guided psychotherapy may play an important role in preventive programs, stepped-care models, integration into primary care, and implementation in low-resource settings where access to mental health professionals is limited [28,34]. In such contexts, these systems may support early detection, psychoeducation, low-intensity interventions, and ongoing symptom monitoring, thereby extending the reach of mental health services.

A key question concerns how AI-guided psychotherapy should be integrated into clinical practice. Current evidence supports hybrid and stepped-care approaches rather than replacement of professionals [6,12]. In these models, clinicians remain responsible for complex cases and higher-intensity interventions, while AI systems provide early support, monitoring, and structured low-intensity care. This approach preserves clinical safety while leveraging scalability.

User experience has emerged as a critical determinant of effectiveness in digital psychotherapy. Outcomes depend not only on adherence to therapeutic principles, but also on usability, perceived responsiveness, and relevance to individual needs [17,18]. Features such as interactivity, personalization, reminders, and supportive language may enhance engagement [8,24,35]. Importantly, many digital interventions fail not because of inadequate therapeutic content, but due to insufficient user engagement and early dropout [18].

One of the key unresolved questions is not whether AI-guided CBT can reduce symptoms, but under what conditions, for which patients, and through which mechanisms these effects occur. While short-term improvements are frequently reported, evidence regarding long-term outcomes and sustained therapeutic change remains limited.

Recent systematic reviews provide an important context for interpreting these findings. Farzan et al. [36] systematically reviewed artificial intelligence-powered CBT chatbots and emphasized their potential for improving accessibility and delivering structured CBT-based support, while also highlighting limitations related to methodological heterogeneity, short-term follow-up, and insufficient evidence regarding long-term clinical effectiveness. Similarly, a recent systematic review in *Archives of Psychiatric Nursing* [37] discussed the integration of AI-powered CBT for autonomous mental health management and underscored both the potential value of these tools for self-directed care and the need for careful clinical oversight, safety procedures, and stronger real-world validation. Compared with these reviews, the present paper contributes by integrating the clinical mechanisms of CBT, digital therapeutic alliance, AI-driven personalization, symptom monitoring, safety considerations, and stepped-care implementation into a clinically oriented conceptual framework for depression and anxiety care.

As discussed, the role of digital therapeutic alliance remains conceptually complex. It is possible that perceived support facilitates engagement, while structured therapeutic exposure and repetition represent the primary mechanisms of symptom improvement.

Finally, successful implementation depends not only on efficacy, but also on system-level factors, including clinician training, integration into existing care pathways, and appropriate regulatory and reimbursement frameworks. These considerations suggest that the future of AI-guided psychotherapy lies in its careful and evidence-based integration into mental health care systems.

Taken together, the available evidence suggests that AI-guided CBT should be positioned within a clinically supervised and ethically governed model of care. Its greatest potential may lie not in replacing existing psychotherapeutic services, but in extending their reach, supporting early intervention, and improving continuity of care for individuals who might otherwise remain untreated.

### 6.1. Digital Therapeutic Alliance

The concept of digital therapeutic alliance requires more critical examination. Digital therapeutic alliance has been increasingly discussed in the recent literature as a potential analog to traditional therapeutic alliance, particularly in the context of digital and internet-delivered interventions [26,27].

While users often report feeling understood, supported, or emotionally validated when interacting with AI systems, it remains unclear whether these experiences reflect a genuine therapeutic alliance or a form of perceived responsiveness shaped by interface design and conversational patterns.

In traditional psychotherapy, therapeutic alliance is a dynamic, reciprocal, and context-dependent process involving shared goals, mutual understanding, and emotional attunement between therapist and patient. In contrast, AI systems operate through predefined algorithms and pattern recognition, which may simulate aspects of empathy without possessing true relational depth or intentionality.

This distinction raises an important question: are improvements in AI-guided CBT driven by a relational process analogous to human therapy, or by functional mechanisms such as repetition, structure, and accessibility.

It is possible that sustained engagement, adherence to structured CBT techniques, and repeated exposure to therapeutic content represent the primary drivers of symptom improvement, rather than a genuine interpersonal alliance. In this context, what is often described as “digital therapeutic alliance” may function more as a facilitator of engagement than as an equivalent of a human therapeutic relationship.

Some studies suggest that perceived alliance in digital interventions may correlate with adherence and outcomes, although findings remain inconsistent and conceptually heterogeneous [26,27].

### 6.2. Mechanisms of Change and Moderators of Treatment Response

Careful consideration should also be given to mechanisms of change. In therapist-delivered CBT, symptom improvement is typically associated with modification of maladaptive cognitions, reduction of avoidance, increased behavioral activation, and the development of adaptive coping strategies [5].

Similar processes may be engaged in AI-guided interventions through self-monitoring, behavioral assignments, structured prompts, psychoeducation, and feedback loops [8,11]. However, digital environments may introduce additional factors—such as usability, immediacy, repetition, and private access—that influence outcomes in ways distinct from traditional therapy settings [18].

The heterogeneity of patient populations represents another important consideration. Depression and anxiety are broad diagnostic categories, and not all individuals are equally likely to benefit from AI-guided interventions. Factors such as symptom severity, digital literacy, motivation, comorbidities, age, preference for self-directed care, and prior treatment experience may all influence treatment outcomes [12,17]. While some users may benefit from the flexibility and anonymity of digital interventions, others may perceive them as insufficiently responsive or emotionally limited. Identifying moderators of treatment response is therefore essential for determining which populations are most likely to benefit, under which conditions, and with what level of human support [18].

### 6.3. Clinical Implementation Within Stepped-Care Models

While Section 5.3 summarizes broad research priorities and unresolved challenges, this subsection focuses specifically on the practical implementation of AI-guided CBT within stepped-care and hybrid clinical models.

The integration of AI-guided psychotherapy into routine mental health care will require an implementation science approach. While initial studies have focused on feasibility and short-term outcomes, broader adoption will depend on factors such as clinician training, reimbursement models, compatibility with clinical workflows, and legal and ethical responsibilities [31].

**Figure 2:**
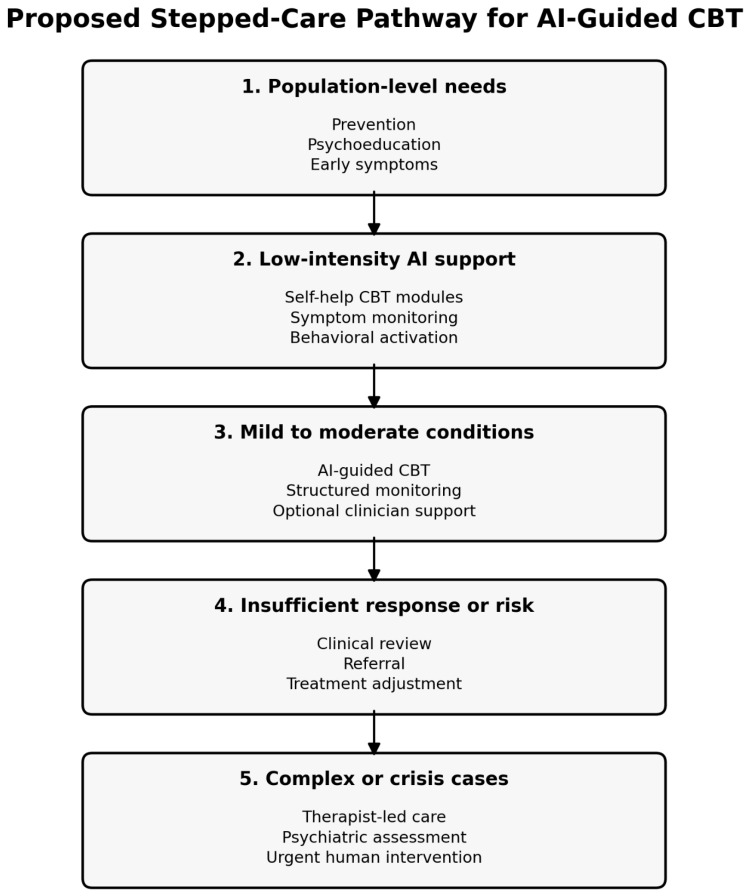
Proposed stepped-care pathway for AI-guided CBT implementation.

A proposed stepped-care pathway for the implementation of AI-guided CBT is shown in Figure 2. Within this model, AI-guided interventions may support prevention, early intervention, and low-intensity care, while clinician involvement remains essential for complex presentations, insufficient treatment response, and safety-related concerns.

Future research should move beyond proof-of-concept designs and focus on effectiveness, feasibility, and sustainability in real-world clinical settings [17]. Protocol-driven studies may play a crucial role in this process by clarifying safety, adherence, and preliminary effectiveness, while also identifying which components of AI-guided care are clinically beneficial and which require human support [12].

Such research is essential for developing evidence-based guidelines and enabling consistent integration of digital mental health interventions across diverse health care systems [33].

In future studies, greater attention should be given to implementation outcomes, including acceptability, feasibility, adoption, fidelity, cost-effectiveness, and sustainability. These parameters are particularly important for determining whether AI-guided CBT can be translated from controlled research settings into routine clinical practice and public mental health systems.

Recent reviews further suggest that implementation research should include standardized outcome measures, longer follow-up periods, transparent reporting of chatbot design features, and clear escalation pathways for users who do not improve or who require human clinical support [20,35].

## 7. Conclusions

AI-guided cognitive behavioral therapy represents a promising, yet still evolving component of modern mental health care. Current evidence suggests that these interventions may improve accessibility and reduce symptoms in selected populations, particularly among individuals with mild to moderate depression and anxiety.

However, important challenges remain, including heterogeneity of interventions, limited long-term outcome data, insufficient real-world validation, and unresolved questions regarding mechanisms of action. In particular, the roles of digital therapeutic alliance, user engagement, AI-driven personalization, and individual differences require further clarification.

This review highlights the importance of understanding AI-guided CBT not only as a technological innovation, but also as a clinically and ethically complex intervention that requires appropriate conceptualization, validation, and governance. The proposed framework emphasizes the interaction between structured CBT mechanisms, user engagement, symptom monitoring, personalization, safety procedures, and public health implementation.

Future research should prioritize rigorous clinical evaluation, mechanism-driven study designs, long-term follow-up, and real-world implementation. Hybrid and stepped-care models, in which AI-guided interventions complement therapist-delivered treatment, are likely to represent the most clinically meaningful and ethically responsible direction.

Rather than replacing mental health professionals, AI-guided CBT should be understood as a complementary and scalable extension of evidence-based psychotherapy. With appropriate clinical validation, ethical safeguards, human oversight, and careful integration into existing health care systems, these approaches have the potential to make a meaningful contribution to reducing the global mental health treatment gap.

## Figures and Tables

**Table 1:** Selected studies on digital and AI-guided CBT for depression and anxiety.

Study	Design	Sample	Intervention	Condition	Key Findings
Andersson et al., 2019 [8]	Meta-analysis	>1000	Internet-delivered CBT	Depression, anxiety	Comparable efficacy to face-to-face CBT in selected populations
Abd-Alrazaq et al., 2020 [11]	Systematic review	Multiple studies	AI chatbots	Mixed mental health conditions	Moderate symptom improvement, limited long-term evidence
Moshe et al., 2021 [18]	Meta-analysis	Multiple studies	Digital interventions	Depression	Significant reduction in depressive symptoms, high heterogeneity
Inkster et al., 2021 [24]	Observational study	Real-world users	AI chatbot	Depression	Improved engagement and self-reported well-being

**Table 2:** Comparison of traditional CBT, digital CBT, and AI-guided CBT.

Feature	Traditional Therapist-Led CBT	Digital CBT/Internet-Delivered CBT	AI-Guided CBT
Mode of delivery	Face-to-face or video-based sessions with a trained therapist	Web-based or mobile structured CBT modules	Interactive digital platform or conversational agent supported by AI
Level of human involvement	High; therapist directs assessment, formulation, intervention, and monitoring	Variable; may be unguided, guided, or therapist-supported	Usually low to moderate; may include automated guidance with optional clinician oversight
Personalization	Based on clinical formulation, therapist judgment, and patient feedback	Usually based on predefined modules and user-selected pathways	Potentially adaptive, based on user input, symptom patterns, engagement, and interaction data
Therapeutic relationship	Direct interpersonal therapeutic alliance between therapist and patient	Limited or mediated therapeutic support, depending on guidance level	Digital therapeutic alliance or perceived responsiveness may support engagement, but does not replace human alliance
Symptom monitoring	Periodic assessment through clinical interview and standardized scales	Repeated self-report assessments and progress tracking	Repeated symptom tracking, interaction-based feedback, and potential risk or progress signals
Core CBT mechanisms	Cognitive restructuring, behavioral activation, exposure, skills training, and relapse prevention	Structured CBT exercises, psychoeducation, behavioral tasks, and self-monitoring	AI-supported delivery of CBT techniques, adaptive prompts, feedback loops, and self-monitoring
Accessibility and scalability	Limited by therapist availability, cost, geography, and waiting lists	More scalable than traditional therapy, especially in guided self-help formats	Highly scalable and continuously available, but dependent on digital access and safe implementation
Main limitations	Workforce constraints, cost, waiting lists, and variable access	Dropout, variable engagement, limited personalization, and heterogeneity in quality	Limited long-term evidence, safety concerns, algorithmic bias, privacy issues, and need for clinical governance
Most appropriate role in care	First-line or specialist treatment, especially for complex or moderate to severe cases	Low-intensity or adjunctive intervention for mild to moderate symptoms	Complementary low-intensity support within stepped-care or hybrid treatment models

## Data Availability

No new data were created or analyzed in this study. Data sharing is not applicable to this article.
